# The Endocannabinoid System as a Window Into Microglial Biology and Its Relationship to Autism

**DOI:** 10.3389/fncel.2019.00424

**Published:** 2019-09-18

**Authors:** Daniel John Araujo, Karensa Tjoa, Kaoru Saijo

**Affiliations:** ^1^Department of Molecular & Cell Biology, University of California, Berkeley, Berkeley, CA, United States; ^2^Helen Wills Neuroscience Institute, University of California, Berkeley, Berkeley, CA, United States

**Keywords:** microglia, endocannabinoids, autism, neuroinflammation, neurodevelopment

## Abstract

Microglia are the resident, innate immune cells of the central nervous system (CNS) and are critical in managing CNS injuries and infections. Microglia also maintain CNS homeostasis by influencing neuronal development, viability, and function. However, aberrant microglial activity and phenotypes are associated with CNS pathology, including autism spectrum disorder (ASD). Thus, improving our knowledge of microglial regulation could provide insights into the maintenance of CNS homeostasis as well as the prevention and treatment of ASD. Control of microglial activity is in part overseen by small, lipid-derived molecules known as endogenous cannabinoids (endocannabinoids). Endocannabinoids are one component of the endocannabinoid system (ECS), which also includes the enzymes that metabolize these ligands, in addition to cannabinoid receptor 1 (CB_1_) and 2 (CB_2_). Interestingly, increased ECS signaling leads to an anti-inflammatory, neuroprotective phenotype in microglia. Here, we review the literature and propose that ECS signaling represents a largely untapped area for understanding microglial biology and its relationship to ASD, with special attention paid to issues surrounding the use of recreational cannabis (marijuana). We also discuss major questions within the field and suggest directions for future research.

## Introduction

Microglia represent a self-sustaining population of cells that originates from the yolk sac and colonizes the brain *in utero* (Alliot et al., 1999; Ginhoux et al., 2010; Bruttger et al., 2015; Askew et al., 2017; Huang et al., 2018). Microglia are the resident immune cells of the central nervous system (CNS) and thus are the first line of defense against CNS infection and injury. For example, they phagocytize debris and pathogens as well as initiate neuroinflammatory responses through release of cytokines such as interleukin-1 (IL-1), IL-6, and tumor necrosis factor alpha (TNF-α) (Yang et al., 2010; Janda et al., 2018). They also recruit natural killer cells, macrophages, and lymphocytes to sites of infection and injury (Yang et al., 2010). Moreover, microglia influence the health of their local environment through release of neurotrophic and neurotoxic factors (Nakajima et al., 2001; Srinivasan et al., 2004; Parkhurst et al., 2013).

In addition to the aforementioned roles, microglia carry out other functions essential for CNS homeostasis (Saijo and Glass, 2011; Butovsky and Weiner, 2018; Lenz and Nelson, 2018). Specifically, these cells oversee neurogenesis by both phagocytizing and directing the migration of newborn neurons (Sierra et al., 2010; Ribeiro Xavier et al., 2015). Microglia also regulate neuronal connections by engulfing excessive synaptic structures through use of the classical complement cascade (Stevens et al., 2007). Lastly, microglia modulate neuronal plasticity via release of neurotrophins such as brain-derived neurotrophic factor (BDNF) (Parkhurst et al., 2013). Early wiring of the brain requires tight control of these processes and therefore microglia critically impact CNS development (Paolicelli et al., 2011; Bialas and Stevens, 2013; Shigemoto-Mogami et al., 2014).

Due to their wide-ranging contributions to CNS homeostasis and development, microglia displaying irregular activity and/or phenotypes can lead to disorders of the CNS (Salter and Stevens, 2017; Butovsky and Weiner, 2018). In this review, we focus on microglial dysfunction as it relates to autism spectrum disorder and associated conditions. We also discuss the role of the endogenous cannabinoid system in modulating microglial involvement in these disorders.

## Microglia and Autism Spectrum Disorder

The Autism and Developmental Disabilities Monitoring Network estimates the current prevalence of autism spectrum disorder (ASD) to be 1 in 59 among children in the United States (Baio et al., 2018). ASD denotes a collection of heterogeneous neurodevelopmental disorders defined by (1) repetitive, restricted behaviors and interests and (2) abnormalities in socio-communication (American Psychiatry Association, 2013). Thus, ASD is an umbrella term and it encompasses several disorders including autism, Asperger’s syndrome, pervasive developmental disorder not otherwise specified, and childhood disintegrative disorder (American Psychiatry Association, 2013). Conditions frequently comorbid with ASD include intellectual disability, attention-deficit/hyperactivity disorder, epilepsy, perturbed sleep patterns, aggression, anxiety, and altered sensory perception (Leitner, 2014; Srivastava and Schwartz, 2014; Fakhoury, 2015; Park et al., 2016; Postorino et al., 2016). These associated conditions can vary in severity and be more or less common within patient subsets. Finally, due to the lack of available therapeutics for ASD, there is a continuous, pressing need for investigation into the causes and progression of the disorder.

Given their role in CNS development and neuroinflammation, microglia are poised to influence the pathogenesis of ASD (Edmonson et al., 2016; Salter and Stevens, 2017; Lenz and Nelson, 2018). Evidence for microglial involvement in ASD comes from both post-mortem- and positron-emission tomography (PET)-imaging studies which show increased neuroinflammation and numbers of activated microglia in brains of ASD patients (Vargas et al., 2005; Morgan et al., 2010; Suzuki et al., 2013). More recently, a large-scale analysis of transcriptomic datasets from post-mortem cerebral cortex has revealed a distinct microglial signature in ASD brains (Gandal et al., 2018). This is concordant with previous observations of microglial activation-related gene enrichment in ASD brain-derived gene networks (Voineagu et al., 2011).

Altered synaptic density is observed in post-mortem ASD brain tissue (Hutsler and Zhang, 2010) and ASD mouse models (Comery et al., 1997; Tang et al., 2014; Wang et al., 2017). These alterations are presumably due to deficits in developmental synaptic pruning (Hansel, 2019). Indeed, current thinking posits that microglia can exert control over the progression of ASD through synaptic pruning dysregulation (Di Marco et al., 2016; Lenz and Nelson, 2018). This hypothesis is supported by the finding that inhibiting microglial autophagy leads to increased synaptic density and reduced sociability in mice (Kim et al., 2017). Moreover, mice with loss of microglia-enriched fractalkine receptor CX3C-chemokine receptor 1 (CX3CR1) display impaired synaptic pruning and reduced social interactions (Zhan et al., 2014). Additional support for the involvement of microglia in ASD pathogenesis comes from studies on mouse models of Rett syndrome (RTT), a syndromic form of ASD caused by mutations in the gene encoding methyl-CpG binding protein 2 (MECP2) (Lombardi et al., 2015). In one model of RTT, neuronal, but not microglial, loss of *Mecp2* leads to excessive synaptic engulfment by microglia in later stages of the disease (Schafer et al., 2016). This suggests that neuronal loss of MECP2 is sufficient to induce aberrant microglial activity and it is consistent with the observation that deletion of *Mecp2* using a *Cx3cr1*-Cre line does not produce RTT-like symptoms (Wolf et al., 2017). Furthermore, *Mecp2-*null microglia produce toxic levels of glutamate that damage post-synaptic structures *in vitro* (Maezawa and Jin, 2010). Still, due to the phenotypic and genetic heterogeneity of ASD, it remains to be seen if these findings in RTT models are representative of autism etiology in general.

Finally, children born to mothers who experience infections or autoimmune disease during their pregnancies are more likely to develop ASD (Jiang et al., 2016; Careaga et al., 2017). This phenomenon, known as maternal immune activation (MIA), has been phenocopied in rodent models (Shi et al., 2003; Patterson, 2011; Careaga et al., 2017; Salter and Stevens, 2017). While embryonic microglia may mediate the neuroinflammatory consequences of MIA (Salter and Stevens, 2017), how this underlies ASD remains unclear.

Considered together, the aforementioned findings implicate microglia as targets for the treatment of ASD. Due to its anti-inflammatory effects, the endogenous cannabinoid (endocannabinoid) system represents a promising tool for modulating microglial involvement in ASD. We next provide a brief overview of the endocannabinoid system and then summarize the evidence linking microglial-endocannabinoid signaling to ASD, with attention paid to issues surrounding the use of recreational cannabis.

## The Architecture of the Endocannabinoid System

**FIGURE 1:**
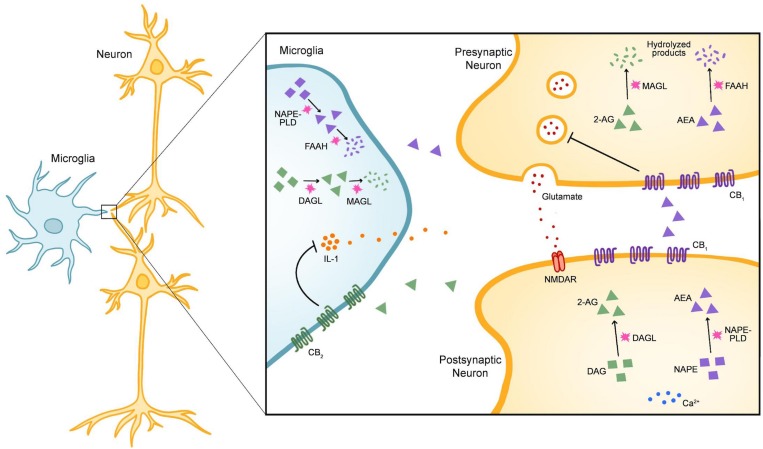
The Components of the Endogenous Cannabinoid System in Microglia and Neurons. In the central nervous system (CNS), the endogenous cannabinoids (eCBs) *N*-arachidonoylethanolamine (AEA) and 2-arachidonoylglycerol (2-AG) are the most widely-recognized ligands of the endogenous cannabinoid (endocannabinoid) system (ECS). The two main receptors for eCBs are cannabinoid receptor 1 (CB_1_) and cannabinoid receptor 2 (CB_2_), both of which are G-protein coupled. Within the CNS, eCB signaling is classically understood to modulate synaptic activity. In the example given here, release of glutamate from presynaptic neurons activates *N*-methyl-D-aspartate receptors (NMDARs) in postsynaptic neurons. In response to increased cytoplasmic calcium, the enzyme diacylglycerol lipase (DAGL) catalyzes the synthesis of 2-AG from diacylgylcerol (DAG) and *N*-acyl-phosphatidylethanolamine phospholipase D (NAPE-PLD) catalyzes the synthesis of AEA from the precursor *N*-acylphosphatidylethanolamine (NAPE). After 2-AG and AEA are released into the synaptic cleft, they stimulate CB_1_ receptors on presynaptic neurons and inhibit further neurotransmitter release. 2-AG is mainly degraded by the enzyme monoacylglycerol lipase (MAGL) whereas AEA is degraded by fatty-acid amide hydrolase (FAAH). While DAGL, NAPE-PLD, MAGL, and FAAH are expressed broadly throughout the CNS, CB_1_ is enriched in neurons and CB_2_ is enriched in microglia. Stimulation of CB_2_ leads to a protective phenotype in microglia that is characterized by a reduction in the release of pro-inflammatory cytokines such as interleukin-1 (IL-1).

The endocannabinoid system (ECS) exerts control over microglial activity and therefore shows promise for treating CNS dysfunction (Benito et al., 2008; Stella, 2009; Lisboa et al., 2016). The ECS consists of three major components: (1) small, lipid-derived endocannabinoids (eCBs), (2) the enzymes responsible for synthesizing and degrading eCBs, and (3) the metabotropic receptors that recognize eCBs (Lutz et al., 2015). The most well-known eCBs in the brain are *N*-arachidonoylethanolamine (AEA or anandamide) and 2-arachidonoylglycerol (2-AG) (Lutz et al., 2015; Parsons and Hurd, 2015). In response to increased cytoplasmic calcium, 2-AG and AEA are synthesized on demand from lipid precursors by the enzymes diacylglycerol lipase (DAGL) and *N*-acyl-phosphatidylethanolamine phospholipase D (NAPE-PLD), respectively (Alger and Kim, 2011; Lutz et al., 2015; Parsons and Hurd, 2015). The enzyme primarily responsible for degrading 2-AG is monoacylglycerol lipase (MAGL), whereas AEA is catabolized by fatty acid amide hydrolase (FAAH). In the CNS, these components are expressed in neurons, microglia, astrocytes, and oligodendrocytes (Figure 1; Stella, 2009; Lutz et al., 2015; Ilyasov et al., 2018). The two main receptors for eCBs include cannabinoid receptors 1 (CB_1_) and 2 (CB_2_), both of which are G protein-coupled (Parsons and Hurd, 2015). Finally, while CB_1_ is enriched in neurons, CB_2_ expression is primarily restricted to microglia (Stella, 2009).

Acute consumption of *Cannabis sativa* (marijuana) yields wide-ranging effects on memory, cognition, appetite, and mood in both humans and rodents (Curran et al., 2016; Kendall and Yudowski, 2016). These effects result from action of the phytocannabinoid (or plant-derived cannabinoid) Δ ^9^-tetrahydrocannabinol (THC) on CB_1_ within the brain (Panagis et al., 2014; Curran et al., 2016; Kendall and Yudowski, 2016). Yet, long-term consequences of cannabis use have been poorly studied, especially with regards to microglia and their impact on neuronal circuitry. Notably, increased eCB signaling is associated with an anti-inflammatory, protective phenotype in microglia (Benito et al., 2008; Stella, 2009; Lisboa et al., 2016). For example, pharmacological inhibition of FAAH decreases microglial activation marker expression, cytokine production, and synaptic plasticity deficits, in the hippocampi of aged rats (Murphy et al., 2012). Additionally, stimulation of CB_2_ inhibits microglial activation and increases striatal neuron survival and motor coordination in a model of Huntington’s disease excitotoxicity (Palazuelos et al., 2009). Moreover, exposure to anti-inflammatory cytokines increases eCB production and CB_2_ expression in microglia (Mecha et al., 2015). These findings cast the ECS as an attractive target for influencing microglial activity (Dhopeshwarkar and Mackie, 2014; Lisboa et al., 2016; Cassano et al., 2017; Donvito et al., 2018). However, the consequences of manipulating eCB signaling on ASD risk and pathogenesis are largely uncharacterized. The increasing legality and use of cannabis currently seen throughout the world therefore requires a better understanding of eCB signaling in microglia as it relates to ASD.

## Microglial-Endocannabinoid Signaling and ASD

### Cannabis Use and ASD Risk

Approximately 2.5–5% of people between the ages of 15–64 years old consume cannabis, making it the most popular illicit drug in the world (Gunn et al., 2016). THC readily crosses the fetal-placental barrier (Wu et al., 2011) and is also secreted in breast milk (Perez-Reyes and Wall, 1982). As of now, there is no strong link between prenatal cannabis use and an increased risk of ASD in offspring. Prenatal exposure to cannabis does correlate with negative outcomes in child development, including growth restriction (Zuckerman et al., 1989; El Marroun et al., 2009; Gunn et al., 2016) and decreased cognitive performance (Richardson et al., 2002; Goldschmidt et al., 2004; Gunn et al., 2016). Still, few studies have been carried out on this topic and these observations are not always reproducible (Wu et al., 2011; El Marroun et al., 2018). Since rates of cannabis use in both pregnant and non-pregnant women are steadily increasing (Brown et al., 2017), there is an unmet need for clarifying the relationship between prenatal cannabis exposure and CNS development. Thus, future clinical and pre-clinical investigations should focus on elucidating the long-lasting effects of prenatal cannabis exposure and if these effects are linked to ASD pathogenesis. Such studies should be paired with efforts to determine if prenatal cannabis exposure impacts microglial synaptic pruning and thereby neurodevelopment in general. These experiments could also take place in the context of pathogen-induced MIA to better recapitulate environmental risks for ASD.

### Cannabinoid Signaling as a Target for ASD Treatment

Pre-clinical evidence supporting the role of ECS signaling in ASD comes from research on rodent models of MIA and neuroinflammation. For instance, in response to the innate immunostimulant polyinosinic:polycytidylic acid [poly(I:C)], MIA-based production of IL-17 induces abnormal cortical development and ASD-like sociability deficits in mouse offspring (Choi et al., 2016). Interestingly, administration of AEA decreases IL-17 production and increases expression of the anti-inflammatory cytokine IL-10 in a mouse model of immune hypersensitivity (Jackson et al., 2014). Treatment with the innate immunostimulant lipopolysaccharide (LPS) during adolescence leads to increased AEA tone and FAAH activity in the amygdala, as well as decreased sociability, in mice (Doenni et al., 2016). These alterations are attenuated with administration of an FAAH inhibitor (Doenni et al., 2016). Still, the contribution of microglia to either the initiation or resolution of these neuroinflammatory effects is unknown and should be the focus of future endeavors.

Cannabidiol (CBD), the second major phytocannabinoid in cannabis (Atakan, 2012), has gained attention as a possible treatment for ASD (Salgado and Castellanos, 2018). Indeed, three clinical reports have recently established that CBD alleviates major symptoms associated with ASD, including seizures, sleeplessness, and anxiety, in children (Barchel et al., 2018; Aran et al., 2019; Bar-Lev Schleider et al., 2019). In addition, because CBD possess a weak affinity for CB_1_ and CB_2_, it has no psychoactive effects and may even prevent some of the harmful consequences of THC (Zuardi et al., 2012; Morales et al., 2017; Mouro et al., 2019). Lastly, a commercially available, oral CBD extract (Epidiolex) has recently gained FDA-approval for treatment of drug-resistant epilepsy (Sekar and Pack, 2019), which can be an additional burden faced by ASD patients (Sansa et al., 2011; Kokoszka et al., 2017; Long et al., 2019). Nevertheless, because exposure to other cannabinoids negatively affects the development of the adolescent brain in rats (Cha et al., 2006; Schneider and Koch, 2007; Quinn et al., 2008), parents and physicians should practice extreme caution when recommending the use of cannabinoids to treat ASD (Atakan, 2012).

Subsequent work in this field must emphasize replicating the usefulness of ECS signaling in ASD via paradigms that include larger and more diverse populations. If these results hold, it will be important to establish if abatement of ASD symptoms is due to eCB signaling in microglia. For example, CBD blocks microglial activation (Martin-Moreno et al., 2011) and neuroinflammation (Elliott et al., 2018; Maroon and Bost, 2018), both of which are linked to seizure susceptibility (Rana and Musto, 2018; Zhao et al., 2018). Consequently, it will be beneficial to establish if CBD-based reduction of epilepsy in ASD patients is reliant on microglial-based mechanisms. Utilizing mice with microglia-specific loss of ECS components in combination with ASD-relevant mouse models could shed light on this area.

## Conclusion

Microglia are indispensable orchestrators of CNS development and homeostasis and are therefore likely involved in the pathogenesis of ASD. Microglial activity can be modulated by eCB signaling, which makes the ECS a potentially forceful tool in the prevention and management of CNS dysfunction. Future work must focus on detailing the mechanisms by which altered eCB signaling in microglia yields protective and detrimental effects in the CNS, particularly as it relates to the effects of chronic cannabis use. Answering these questions could provide improved therapeutics for ASD and its associated conditions.

## Author Contributions

All authors wrote the manuscript. KT and DA designed the figure.

## Conflict of Interest Statement

The authors declare that the research was conducted in the absence of any commercial or financial relationships that could be construed as a potential conflict of interest.
